# Structural and wetting properties of nature's finest silks (order Embioptera)

**DOI:** 10.1098/rsos.180893

**Published:** 2018-09-12

**Authors:** Grace Y. Stokes, Evangelea N. DiCicco, Trevor J. Moore, Vivian C. Cheng, Kira Y. Wheeler, John Soghigian, Richard P. Barber, Janice S. Edgerly

**Affiliations:** 1 Department of Chemistry & Biochemistry, Santa Clara University, 500 El Camino Real, Santa Clara, CA 95053, USA; 2 Department of Physics and Center for Nanostructures, Santa Clara University, 500 El Camino Real, Santa Clara, CA 95053, USA; 3 Department of Biology, Santa Clara University, 500 El Camino Real, Santa Clara, CA 95053, USA; 4 Department of Environmental Sciences, The Connecticut Agricultural Experiment Station, New Haven, CT, USA

**Keywords:** silk, Embioptera, webspinners, embiids, contact angle, water adhesion

## Abstract

Insects from the order Embioptera (webspinners) spin silk fibres which are less than 200 nm in diameter. In this work, we characterized and compared the diameters of single silk fibres from nine species—*Antipaluria urichi*, *Pararhagadochir trinitatis*, *Saussurembia calypso*, *Diradius vandykei*, *Aposthonia ceylonica*, *Haploembia solieri*, *H. tarsalis*, *Oligotoma nigra* and *O. saundersii*. Silk from seven of these species have not been previously quantified. Our studies cover five of the 10 named taxonomic families and represent about one third of the known taxonomic family-level diversity in the order Embioptera. Naturally spun silk varied in diameter from 43.6 ± 1.7 nm for *D. vandykei* to 122.4 ± 3.2 nm for *An. urichi.* Mean fibre diameter did not correlate with adult female body length. Fibre diameter is more similar in closely related species than in more distantly related species. Field observations indicated that silk appears shiny and smooth when exposed to rainwater. We therefore measured contact angles to learn more about interactions between silk and water. Higher contact angles were measured for silks with wider fibre diameter and higher quantity of hydrophobic amino acids. High static contact angles (ranging up to 122° ± 3° for *An. urichi*) indicated that silken sheets spun by four arboreal, webspinner species were hydrophobic. A second contact angle measurement made on a previously wetted patch of silk resulted in a lower contact angle (average difference was greater than 27°) for all four species. Our studies suggest that silk fibres which had been previously exposed to water exhibited irreversible changes in hydrophobicity and water adhesion properties. Our results are in alignment with the ‘super-pinning’ site hypothesis by Yarger and co-workers to describe the hydrophobic, yet water adhesive, properties exhibited by webspinner silk fibres. The physical and chemical insights gained here may inform the synthesis and development of smaller diameter silk fibres with unique water adhesion properties.

## 1. Introduction

New technologies are being developed to synthesize artificial fibres with unique physical, mechanical or water-interaction properties based on genetically modified silk proteins from spiders and silkworms [1]. Naturally spun fibres from these arthropods are of the order of 1 to 10 µm in diameter [2]. Technological advances warrant an interest in preparing smaller diameter (finer) fibres, which may be used in nanoscale medical or optical devices [3]. To inform the synthesis of finer silk fibres, developers may take inspiration from insects of the order Embioptera (commonly called webspinners or embiids), which spin nature's ‘finest known insect silk fibres’ [4]. Physical and chemical properties of webspinner silk for only five species have been published [4–9]. In the current work, we characterize and compare the diameters of single silk fibres from nine species. The species in this study provide a range of evolutionary histories, body sizes and lifestyles, from arboreal to detritivores. We established experimental methods for determining the variability of silk fibre diameter within the order Embioptera and explored interactions between silk and water. Given our unique access to a number of different species, we had the opportunity to test two hypotheses regarding single fibre diameters: (i) larger webspinners yield larger diameter silk (as the ‘finest known silk’ comes from a very small webspinner [4]); and (ii) silks from evolutionarily closely related webspinners have more similar diameters.

### 1.1. Natural history of webspinners (class Insecta, order Embioptera)

**Figure 1.**
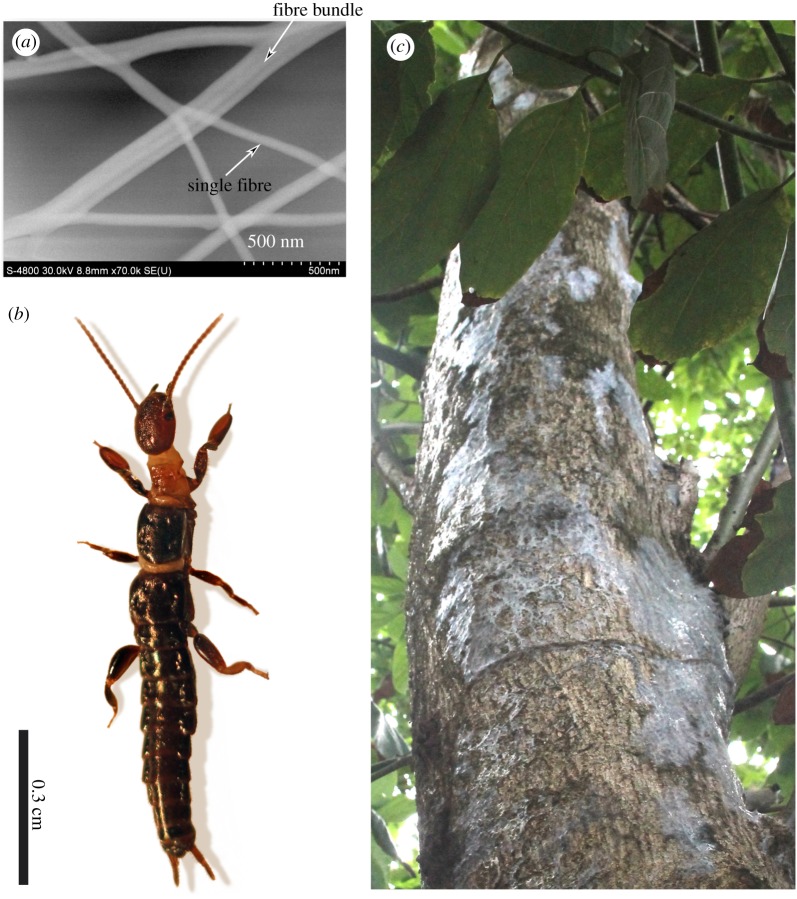
(*a*) SEM image of *Pararhagadochir trinitatis* naturally spun silk fibres with aluminium coating—note the difference in diameter of fibre bundles and single fibres. (*b*) Adult female *P. trinitatis*. (*c*) *Pararhagadochir trinitatis* silk in its natural environment—note the shiny patches apparently due to interactions with rainwater.

Webspinners are mostly tropical and subtropical in distribution and spin by secreting nanoscale fibres (figure 1*a*) from silk glands housed in their front feet [10]. All individuals (male, female, immature and adult) spin by stepping with their front feet against the substrate while releasing multiple strands of silk from enlarged tarsal glands. The webspinners are soft-bodied and flexible, and the females are always wingless as shown in figure 1*b*; these features allow them to easily run backwards and forwards within their tightly spun silken tunnels. With stereotypical stepping patterns [11], they create protective silken domiciles on trees (figure 1*c*) in humid climes. In dry regions, they live in leaf litter or in underground burrows, which they line with silk. Species used in this survey were from five of the 10 named taxonomic families in the order Embioptera: *Antipaluria urichi* (family Clothodidae), *Pararhagadochir trinitatis* (family Scelembiidae) and *Saussurembia calypso* (family Anisembiidae), all from Trinidad in the West Indies; *Diradius vandykei* (family Teratembiidae) from the southern USA; and five species in the family Oligotomidae: *Aposthonia ceylonica* from India, *Haploembia solieri* and *H. tarsalis* (introduced into California from the Mediterranean region) and *Oligotoma nigra* and *O. saundersii* (pantropical and introduced throughout warmer regions of the world). Our survey included representatives from about one third of known families of webspinners [12].

### 1.2. Measuring silk fibre diameter without a metal coating

To quantify silk diameters, previous scanning electron microscopy (SEM) analyses of webspinner silk fibres used metal coatings to prevent sample charging [4,5,12]. For a fibre that is 30–100 nm in diameter, a 10–20 nm coating can represent a significant error in size measurements, especially in the context of comparing fibres from different species. We are unaware of any reports of SEM images of webspinner silks in the absence of a metal coating, except for transmission electron microscopy (TEM) images which also changed the fibre diameter through chemical manipulation of the silks (uranyl acetate caused the fibre to swell) [5]. Additionally, in order to understand how exposure to water changes the morphology or structure of the fibres, depositing metal may obscure what we wish to observe. In this study, webspinners were allowed to spin silk directly onto solid cylindrical graphite rods, which were then used in SEM analyses. The use of graphite rods was inspired by the sample preparation used in a study of spider silk [13]. Tension in the threads tended to pull them towards the surface thereby improving contact. The sufficient conductivity of the graphite allowed us to make clear images of single fibres, therefore eliminating the need for a metal coating.

### 1.3. Interactions with water (wetting and adhesion)

The shiny patches visible on the silk in figure 1*c* aroused our curiosity and led to an exploration of how rain may trigger a transformation of the otherwise fibrous, cloth-like nature of webspinner silk. Four arboreal species from four families (*An. urichi* (Clothodidae), *P. trinitatis* (Scelembiidae), *Ap. ceylonica* (Oligotomidae) and *S. calypso* (Anisembiidae)), were chosen because they share similar habitats, which experience heavy tropical rains, a situation that potentially imposes selective pressures on the insects to produce effective silk coverings. A webspinner silk fibre is thought to be composed of a lipid coating surrounding a protein core [5,6]. Without this lipid coating, the silk fibre was observed with TEM to be permeable to water [6]. Previous NMR studies described how organic solvents (2 : 1 chloroform/methanol) changed the hydrophobicity and permeation of webspinner silks to water [5,6]. In the current study, we evaluate whether in the absence of organic solvents, exposure of webspinner silk to water can also change its water permeability properties and macroscopic structure.

A previous study of *An. urichi* silk included contact angle measurements, which indicated that the silk was superhydrophobic (water drops bead up on it) but also highly water adhesive (water drops do not roll off) [6]. Silken sheets were composed of sparse, unevenly spaced fibres, which were laid ‘down in layers’ and not uniform in thickness [6]. High advancing contact angles of 150° ± 2° and low receding contact angles (approaching 0°) were measured [6]. High contact angle hysteresis and adhesive quality have also been previously quantified for the surfaces of rose petals [14,15]. This ‘rose petal effect’ was attributed to water's inability to penetrate into the uniformly distributed nanoscale features on microscale ‘bumps’ on the surface of a rose petal. Osborn Popp and co-workers suggested that lipid coating on webspinner silk was ‘only mildly hydrophobic’, and that the ‘rose petal effect’, was not relevant to silk fibres, which were ‘loosely woven’ and not uniform [6]. As the water droplet volume decreased when the receding contact angle was measured, fibres were drawn together, forming an aggregated pinning site, which adhered water to the silk surface (see fig. 6 in [6]). The hydrophobic and adhesive qualities of webspinner silk were attributed to these ‘super-pinning’ sites. In the current work, we extend these studies to include more species. We measure both static contact angles and tilt angles, motivated by previous studies of water interactions with flower petals and replicas [14].

## 2. Experimental section

### 2.1. Collection and preparation of embioptera silk

Nine species were available to conduct comparative studies of their silk. *No insects were euthanized or harmed for this project*. The insects are easily reared, for the most part, in boxes filled with dried live oak leaves that form a matrix for their silk galleries. Silk was collected from the boxes or jars, often containing hundreds of individuals having been maintained continually for many years at Santa Clara University. They were fed romaine lettuce or lichens and misted with water twice a week. The two *Haploembia* species were not kept in large laboratory populations but rather were collected in the field when needed. These two species do not thrive in culture over the long term and thus were maintained in smaller groups where they could spin on the apparatus needed for the SEM work. The species varied in ecology: three leaf-litter species (*H. solieri*, *H. tarsalis* and *O. nigra*) and six arboreal species (*An. urichi*, *P. trinitatis*, *S. calypso*, *D. vandykei*, *O. saundersii* and *Ap. ceylonica*).

Silk samples selected for this analysis were spun by the insects, held in large culture boxes, onto dried live oak leaves. We have three different collection methods:

(1) For silk used to measure tilt angle, we allowed the insects to spin on a live oak leaf. These leaves are stiff and curved in such a way that the sheet-like silk is suspended above the substrate, providing a space for a domicile. The configuration of each sample meant that water droplets touched silk, not the oak leaf below.
(2) For silk used to measure fibre diameter, we exposed the substrate materials (graphite rods of two sizes—2.4 and 6.4 mm diameters) to small groups of adult females and late instar nymphs in Petri dishes. See §2.2 below for rationale for using graphite rods.
(3) For silk used to measure contact angle, we needed cloth-like patches. We lifted out these patches from a colony for each species and suspended the silk across a notched space in a Plexiglas holder to ensure water droplets would touch only silk.

Although previously published accounts of *An. urichi* silk placed on glass slides revealed superhydrophobic contact angles [6], when we allowed insects from *An. urichi*, *Ap. ceylonica* and *P. trinitatis* species to spin silk on glass slides in preliminary trials, we measured lower initial contact angles. We concluded that the hydrophilic quality of glass interacted with water, leading us to suspend the silk samples. Subsequent trials showed higher initial contact angles compared to silk on glass (analysis of variance, ANOVA; *F*_5,30_ = 10.16; *p* = 0.0033), further supporting our decision to test only suspended silk. For this and all subsequent statistical tests, we used the software package JMP Pro 13 (v. 13.1) by SAS Institute (Cary, NC).

### 2.2. Scanning electron microscopy

**Figure 2.**
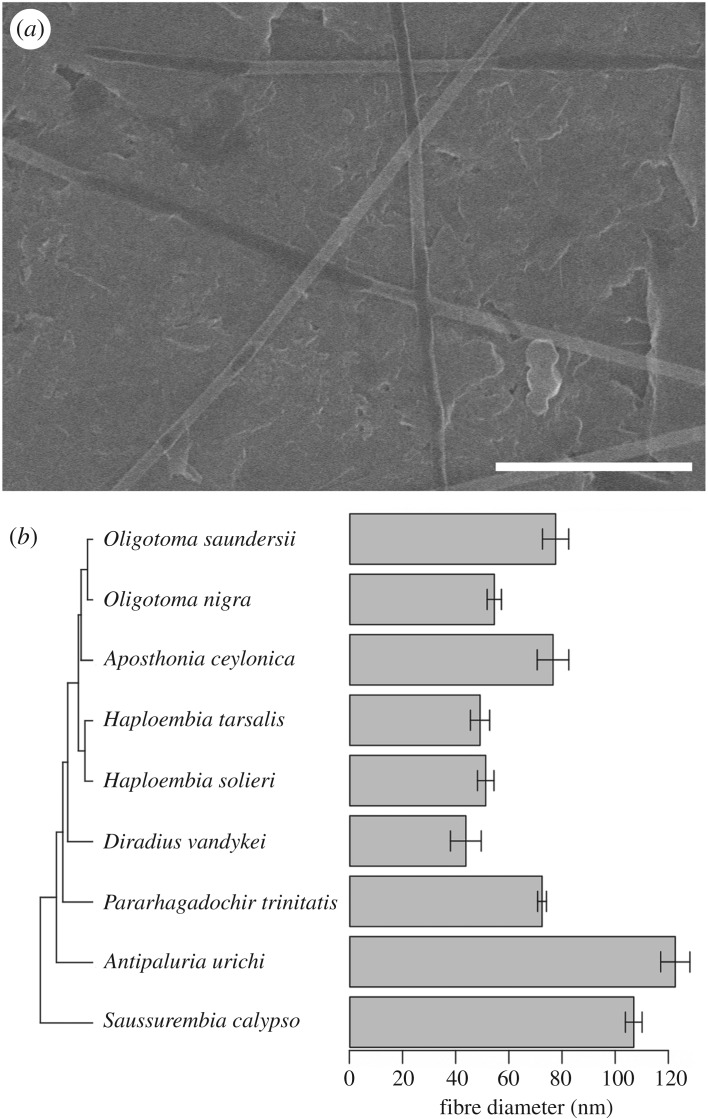
(*a*) SEM image of *Pararhagadochir trinitatis* fibres as spun onto a graphite rod. The scale bar designates 1 μm. (*b*) Fibre diameter (mean ± s.e.) determined from SEM images of silk spun onto graphite rods by nine species. Phylogenetic tree aligned along the horizontal axis shows the evolutionary relationships between these species.

All SEM images were produced using a Hitachi S-4800 field emission scanning electron microscope. Diameters of single silk fibres were determined from images of silk from nine species. An example of an SEM image of a non-metallized sample is shown in figure 2*a*. The number of samples used for each species is shown in electronic supplementary material, table S2. Good SEM imaging requires that samples can conduct the electron beam current to ground. In the case of poorly conductive webspinner silk, previous approaches sputter-coated the samples with up to 10–15 nm of gold [4,5]. Silk fibres on the graphite surface are able to shed the electron beam current well enough for good imaging without a metal coating. The difficulty of this technique is that fibres that are not close to the surface suffer charging effects and cannot be reasonably imaged. In order to reduce charging, the SEM was operated at 15 kV with a current of 5 µA (lower than the typical 10 µA beam current). With diameters observed down to 30 nm, this technique should result in better accuracy compared to those that coat fibres with tens of nanometres of metal.

**Figure 3.**
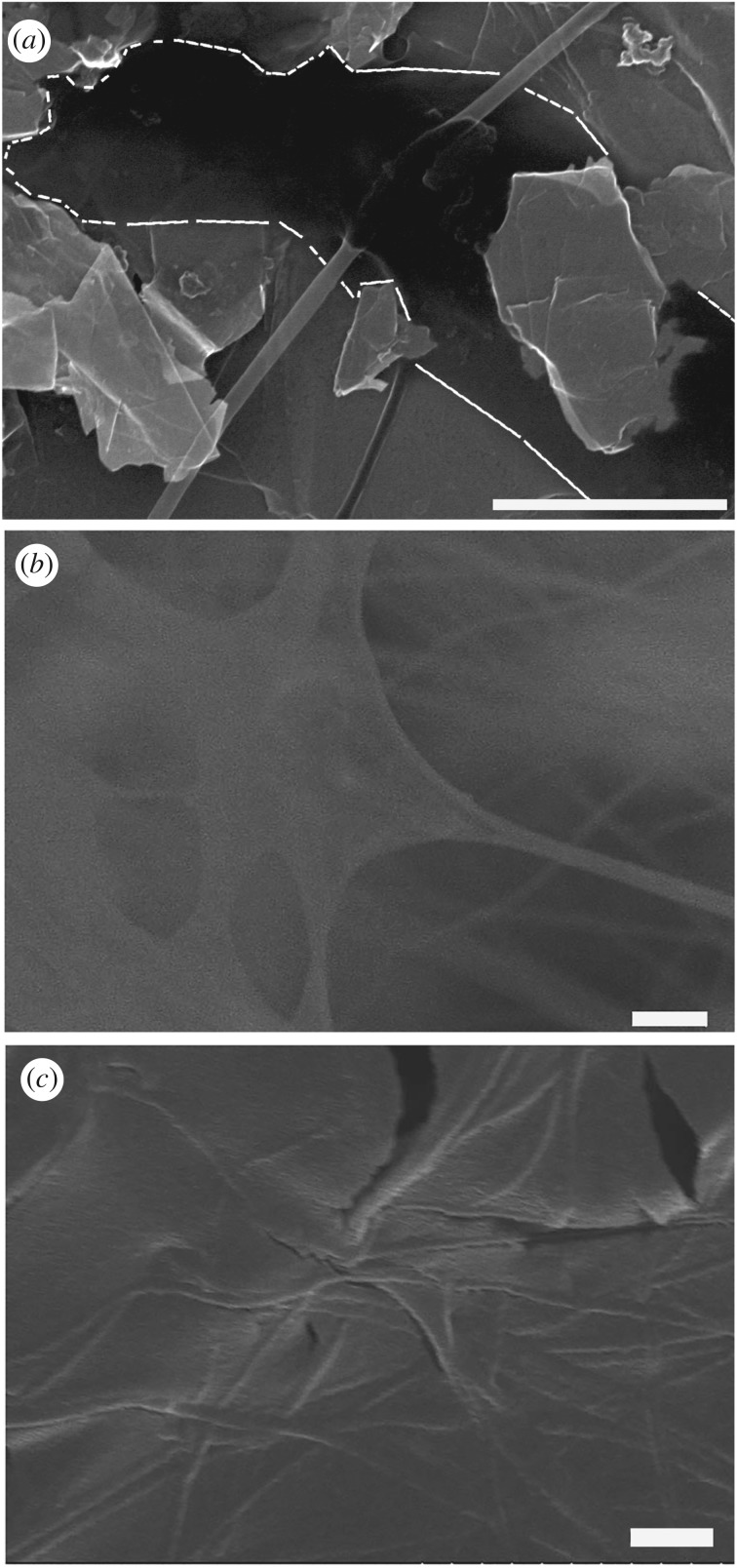
SEM images of (*a*) one single *Antipaluria urichi* fibre on a graphite rod that was previously wetted—note the dark region is outlined with dashed white line indicating the residue from the dissolved protein core (see manuscript for details), (*b*) a region of *P. trinitatis* ‘film’ formed from dried fibre core residue—note the apparent remnants of silk fibres supporting this film (in order to reveal the underlying structure this sample was not metallized, leading to a poorer image quality), and (*c*) an example of a metallized *P. trinitatis* film where the fibre remnants are visible and in this case not obscured by the coating. The scale bars all designate 1 μm.

SEM imaging of silken sheets, composed of bundles of single fibres heterogeneously woven together, was performed on samples coated with 20–30 nm of aluminium (Al). These samples were prepared by thermal evaporation in a vacuum of less than 0.8 × 10^−6^ mbar. In certain cases, the insects were allowed to spin onto dry live oak leaves that had been previously metallized. The samples were then metallized again to provide adequate electrical conduction to ground. An example of an SEM image of metallized sample is shown in figure 3*c*, and is discussed in detail in §3.1 below.

SEM was used to determine the changes in nanofibres in response to wetting. Using a syringe, small amounts of deionized 18 MΩ cm water were applied to both graphite rods bearing single fibres and to silken sheets spun onto dry live oak leaves. We refer to these samples as ‘previously wetted.’ SEM images were acquired for non-metallized samples of both previously wetted single fibres and silken sheets; however, low conductivity produced relatively poor images of the latter. More frequently, metallization was used to produce sharper images for the previously wetted silken sheets.

### 2.3. Phylogenetic analysis of fibre diameter

The relationship between fibre diameter and adult female body length (see electronic supplementary material, table S2 for means ± s.e.) across the order Embioptera was evaluated using linear models. As species are not independent statistical units, and share evolutionary history that could influence a relationship between body size and fibre diameter, models that account for the evolutionary relatedness of species (so-called phylogenetic least squares) were used and compared with traditional ordinary least squares. Such phylogenetic analyses require a phylogeny of the taxa in question. As such, the maximum-likelihood phylogeny for Embioptera in this study was inferred from a concatenated multisequence alignment previously published in Miller *et al*. [12]. Our analysis relied on GenBank sequences as reported in electronic supplementary material, table S1 of Miller *et al*. [12]; the codes noted below indicate which samples were used in our phylogenetic tree for the species of our study. The 7349 base pair alignment comprised partial sequences of the mitochondrial 16S and CO1 regions, and nuclear 18S, 28S, and H3 regions (reported by Miller *et al*. [12]). The alignment was trimmed to include *O. nigra* (EB1 in the original alignment), *O. saundersii* (EB50), *H. tarsalis* (EB29), *H. solieri* (EB93), *D. vandykei* (EB37), *S. calypso* (EB36), *An. urichi* (EB9), and *P. schadei* (EB25), plus two outgroups, *Blatta orientalis* and *Tyrannophasma gladiator*. The alignment was partitioned according to gene regions, and substitution models for each gene were inferred by ModelFinder [16] in IQ-Tree [17]. The maximum-likelihood phylogeny was then inferred using these substitution models in IQ-Tree with an Ultrafast Bootstrap analysis and default settings.

All phylogenetically informed analyses were performed in R version 3.4.1 [18]. The resulting maximum-likelihood phylogeny was rendered ultrametric for subsequent comparative analyses using penalized likelihood and the function *chronos* from the R package ape [19]. Because there was no sequence data available for *P. trinitatis*, we used branch length information from the closely related *P. schadei* in place of *P. trinitatis* in all analyses. The phylogenetic signal of average body length and average fibre diameter for each species was evaluated using Blomberg's K as implemented in the function *phylosig* from the R package phytools [20]. All linear models used average fibre diameter per species as the dependent variable and average body length per species as the independent variable. Phylogenetic linear models were estimated using the phylolm package [21], accounting for evolutionary relationships under the stochastic Brownian motion process, as well as under an Ornstein–Uhlenbeck (OU) process, which models natural selection acting on a single optimum [22].

### 2.4. Contact angle hysteresis

In order to assess variation and degree of hydrophobicity of silk from the four arboreal species, silk samples were assayed using a First Ten Angstroms (FTÅ 1000B) goniometer, providing quantification of the contact angle that forms between a droplet of deionized 18 MΩ cm water and a substrate. ANOVA and matched pairs *t*-test were used to test for differences between contact angles while applying a criterion of *p* < 0.05 for significance. For comparison, initial contact angle of water droplets (*n* = 5) placed on a sample of polydimethylsiloxane (PDMS) was measured to serve as a hydrophobic standard. Similarly, initial contact angle of water droplets (*n* = 5) on a piece of a rose petal collected on the Santa Clara University campus and affixed to a glass slide was measured to serve as a model for superhydrophobicity [23]. Initial contact angles were measured on 15 silk samples each for *An. urichi*, *Ap. ceylonica* and *P. trinitatis* and on 14 samples for *S. calypso*.

**Figure 4.**
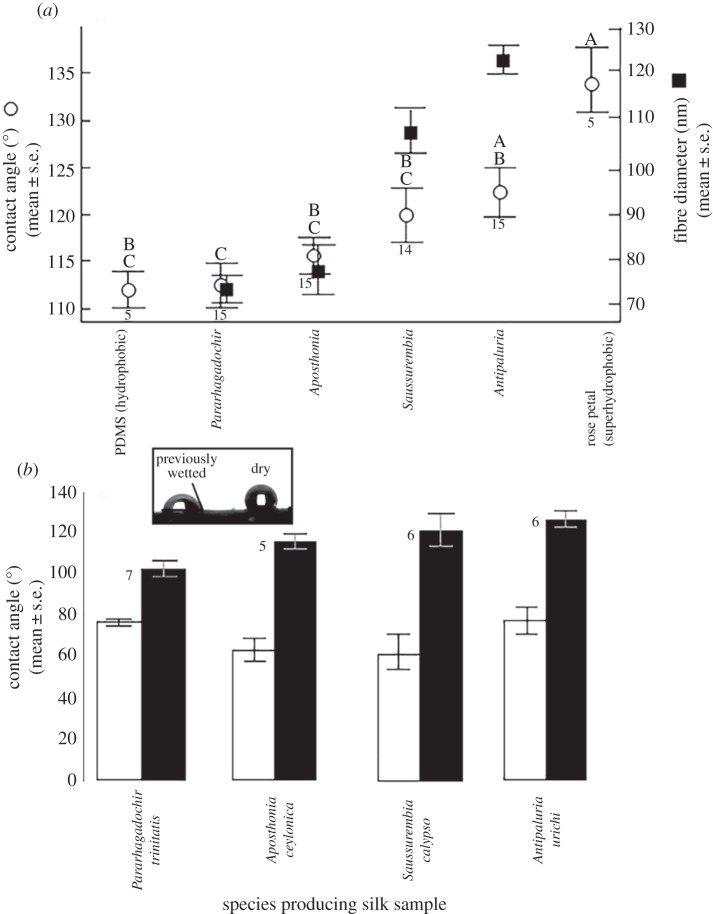
(*a*) Mean ± s.e. of static contact angles (open circles, graphed on left axis) and mean ± s.e. of fibre diameters (filled squares, graphed on right axis) from SEM images of silk from four arboreal, tropical webspinner species. For fibre diameter measurements, sample sizes for each species are shown in electronic supplementary material, table S2. Comparisons were made to PDMS and rose petal. Different upper-case letters (A, B or C) above each bar indicate significant differences in contact angles based on the results of an analysis of variance. Where these letters are the same, the samples are considered not statistically different. For example, average contact angles for rose and *An. urichi* are not considered statistically different, and thus the letter A appears above both values. All other silk samples yielded contact angles different from rose. Numbers below each bar represent sample size for contact angle measurements. (*b*) Static contact angles (mean ± s.e.) versus species for both dry (right) and previously wetted (left) silk. Numbers above each bar represent sample size. INSET: water droplet on dry (right) and previously wetted (left) *Ap. ceylonica* silk.

To determine if previously wetted, air-dried silks interact differently with water, contact angles were measured for adjacent regions—one dry and one previously wetted with a 5 µl drop of DI water and air-dried—on the silk samples. A representative photograph of this set-up is shown in an inset in figure 4*b*. The previously wetted region appeared as a shiny spot on the silk, providing a target for us while working with the goniometer. The shiny spot emulated the sheen previously detected on silk found in the tropical forest in Trinidad (figure 1*c*). During testing, one 1–2 µl droplet was placed on the suspended silk samples for each of two regions, yielding statistically paired samples. The number of paired samples were as follows*: An. urichi* (*n* = 5), *P. trinitatis* (*n* = 3), *Ap. ceylonica* (*n* = 4) and *S. calypso* (*n* = 3).

### 2.5. Tilt angles

Following an analogous protocol to the one described above, ANOVA and matched pairs *t*-test were used to test for differences between tilt angles of silk fibres from the same four species, and before and after exposure to water. Tilt angle (TA) measurements are used to quantify adhesive properties of materials by determining the angle of incline at which an applied water droplet falls from a substrate. This method provides a way for us to assess and compare adhesive qualities of silks from the four species also tested for contact angle. TA has also been used to describe the adhesive quality of the ‘rose petal effect’, and our TA methods emulated those used to test adhesiveness of flower petals [14,23].

During testing, each silk-leaf sample was firmly taped onto a flat platform designed to rotate and in such a manner that the silk sheet was flat and parallel to the platform. A photograph of the experimental apparatus is found in the electronic supplementary material, figure S3. Water droplets were added in increments of 10 µl until the volume reached 50 µl. Once 50 µl was reached, allotments of 5 µl were added until the drop slipped off the sample during rotation. With each addition of volume, the sample was completely rotated at a speed of 26.5° per second through 360°. The volume at which the droplet fell and the TA were recorded for three samples of dry silk (four for *An. urichi*) and three samples of previously wetted, air-dried silk for each species. Digital video recordings were made of each trial and the angle of tilt at the moment the droplet slipped off was calculated using ImageJ [24] during video playback on an iMac Computer. Volume of the droplet that fell and the TA were compared between dry and previously wetted silks as a function of species using ANOVA.

### 2.6. Infrared spectroscopy methods

Attenuated total reflectance–Fourier transform infrared spectra (ATR-FTIR) were collected using a Shimadzu IRAffinity-1S spectrophotometer equipped with a single reflection ATR accessory (Specac Quest P/N GS10800). For each sample, silk bundles were pressed into the diamond window with the ATR anvil and 20 scan averages were collected after a background scan. The diamond window and anvil were both cleaned with isopropanol and methanol on a Kimwipe in between each sample.

## 3. Results and discussion

### 3.1. Scanning electron microscopy images of dry silks

#### 3.1.1. Quantification of fibre diameter using metal-free samples

Our motivation for developing a metal-free technique for measuring fibre diameters was to remove the inherent uncertainty of adding thicknesses of the same order of magnitude as the samples. Since both the deposition of these metallic layers and the observation by the SEM are directional, it is unclear exactly how to treat subtracting these added thicknesses in order to derive the correct silk diameter. With respect to the location of the sample, the points of view of the deposition source and the SEM are not easily known, so a reliable method for correcting for the added 10–20 nm of metal is not obvious. This detail is especially challenging when measuring fibres with diameters as low as 30 nm. We had a small set of Al-coated fibre diameters that we compared to uncoated ones. The average fibre diameters measured for *An. urichi* were 100 ± 33 nm (uncoated) and 160 ± 54 nm (Al-coated). These values are consistent with the 10–20 nm added Al thickness. Furthermore, our results are consistent with previous data [5], indicating that subtraction of estimated metal thickness can be an acceptable approach for larger diameter samples. However, in undertaking a large survey of silk diameters from nine species using many images, our metal-free approach has the significant advantage of eliminating a source of variability from sample preparation. This improvement is especially important for the smallest diameter fibres. A previous study vacuum dried samples from *H. solieri* before metallization [12]. Our measurements for the same species are comparable to or slightly smaller than theirs even though the thickness of their gold coating was not specified. All samples in our studies and in the studies in [12] were SEM imaged under vacuum. We may assume that placing natural silk under vacuum has no significant effect on fibre diameter because significant swelling in our samples due to additional water would suggest that our samples should be larger (not comparable or smaller) compared to the twice ‘dehydrated’ samples. The narrower or comparable diameters of our metal-free *H. solieri* samples are consistent with the previously reported diameters of metal-coated silk fibres in [12].

An SEM image of *P. trinitatis* fibres as spun on a graphite rod is shown in figure 2*a*. Regions of the fibres that are in good electrical contact with the substrate appear with dark centres, whereas poor contact leads to a brighter glow. This image is a good example of those used for determining the diameter data in figure 2*b*.

#### 3.1.2. Fibre diameters of naturally spun dry silk vary with phylogenetic relatedness

The fibre diameters measured for each of nine webspinner species using metal-free samples are shown in figure 2*b*. The species are grouped by phylogenetic tree because a statistically significant phylogenetic signal in fibre diameter between species was observed (*K* = 1.18, *p* = 0.02). In other words, species that were more closely related had generally more similar silk fibre diameters. For example, the two *Haploembia* species in this analysis are similar in fibre diameter, even though *H. solieri* is smaller than *H. tarsalis* as an adult female (electronic supplementary material, figure S1). Adult female body length did not show phylogenetic signal (*K* = 0.80, *p* = 0.20). Moreover, the average adult female body length was not significantly associated with average fibre diameter in an ordinary least-squares linear model (intercept *t* = 0.47, *p* = 0.67; slope *t* = 1.72, *p* = 0.13); or when accounting for phylogenetic relatedness under models of Brownian Motion (intercept *t* = 1.28, *p* = 0.25; slope *t* = 0.93, *p* = 0.39) or an Ornstein–Uhlenbeck process (intercept *t* = 0.75, *p* = 0.48; slope *t* = 1.52, *p* = 0.17). Thus, from our dataset, it appears species relatedness explains more variance in fibre diameter than does average adult female body length of webspinner species.

#### 3.1.3. Scooped versus spun silks

Both sets of silk diameters (scooped and naturally spun) show significant differences depending on the species that produced the silk. As shown in electronic supplementary material, figure S2, scooping the silk and wrapping it onto the graphite rod tended to obscure the differences in diameter and resulted in more overlap between fibre diameters for the different species. For example, *An. urichi* scooped silk was 72.3 ± 8.2 nm (s.e.) on average compared to 122 ± 3.5 nm when naturally spun. Naturally spun silks revealed more variability between species for silk diameter. Scooping the silk had a homogenizing effect on the silk fibres, but the specific effect on the silk was variable (half the samples showed an increase in diameter, the other half a decrease compared to natural silk). One explanation for this variability is that fibres could either be stretched or relaxed in the process of scooping. The fibre diameters determined from naturally spun samples are thought to be more accurate because SEM image quality was higher because the insects had stuck the fibres to the surface, thereby producing both a *natural* tension and enhanced electrical conduction. For both of these reasons, we present only the as-spun silk results in the main text and include data from scooped samples and the statistical analyses in the electronic supplementary material.

#### 3.1.4. Scanning electron microscopy images of previously wetted, naturally spun silk

In order to elucidate the mechanisms for film formation in wetted silk (see figure 1*c* for the naturally occurring structures), we obtained SEM images of previously wetted fibres. These images show that previously wetted silk exhibits a film-like quality. Figure 3*a* shows a single *An. urichi* fibre from a previously wetted sample. Although the entire area shown in the image was exposed to water (wetted on a macroscopic scale), only part of the fibre appears to have been affected. The darker region in the centre of the image is identified as the residue from the soluble protein core. This image suggests that water was able to penetrate the lipid coating and dissolve the protein core, but only in a localized section of the fibre. Our confidence in this interpretation comes from observing over one hundred images of dry single fibres on graphite. We can easily discern silk and the small graphite crystallites, and we never observe darkened regions as shown in figure 3*a* until we expose the silk to water. Figure 3*a* is representative of dozens of previously wetted samples of single fibres. It is important to note that these regions only appear in proximity to the silk fibres, and when they do appear, they appear in abundance. Furthermore, we did comparative imaging of a clean graphite rod before and after the same wetting treatment as used with silk-bearing rods. We observe no difference in the images from identical locations on the surface, and no comparable darkened regions appear. The only reasonable explanation for this darker region is the presence of residue from the silk. This effect has been observed in multiple previously wetted samples.

A suspended film of *P. trinitatis* silk is presented in figure 3*b*. It appears that an underlying silk layer forms a scaffold underneath the previously wetted silk. The absence of a metal layer makes it possible to see the underlying lattice presumably formed from the outer lipid layer of the fibres. This image has inferior clarify because the sample is not in good electrical contact with ground (the structure is not adhered to the graphite *and* there is no metallization). Figure 3*c* is an example of a metallized previously wetted *P. trinitatis* silk film, which has better image quality. In this case it is also possible to see *some* of the underlying lattice of fibres even with the metallic layer.

Our observations of the interaction of water with both the single fibre samples (figure 3*a*) and larger structures (figure 3*b,c*) suggest a new model for the structural transformation in the wetted silk samples. The protein core of the silk is apparently dissolved by the water that is adhered to the silk. As this water evaporates, the residual protein exhibits a new morphology as a film suspended between the fibre remnants. The structural transformation accounts for the shiny sheet appearance observed in the field (figure 1*c*). To further elucidate transformations due to interactions with water, contact and tilt angles were measured.

### 3.2. Contact and tilt angles

#### 3.2.1. Contact angle hysteresis

Initial contact angles varied significantly, but with extensive overlap, between silk samples depending on species (figure 4*a*; *F*_5,63_ = 5.976; *p* < 0.0001). *An. urichi* silk generated statistically higher contact angles on average (122° ± 3° (s.e.)) compared to *P. trinitatis* silk (112° ± 2°). The contact angles of the other two species were intermediate, overlapping statistically with both *An. urichi* and *P. trinitatis*. Water placed on *An. urichi* silk behaved similarly to water on rose petals; these values approached superhydrophobicity (greater than 150°). Despite the higher average for *An. urichi*, silks of all four embiopteran species interacted with water in a statistically similar manner to the hydrophobic PDMS. Variability was most likely caused by the natural variation generated by the insects spinning in their laboratory containers (refer to electronic supplementary material, figure S5 as an example) and by our sampling method of scooping silk samples onto the notched Plexiglas holders.

Contact angles of paired droplets on treated silk samples were significantly different: previously wetted, air-dried silk became hydrophilic while adjacent dry spots remained hydrophobic, irrespective of the species producing the silk (figure 4*b*; matched pairs *t*-test = 7.99; *p* < 0.0001). Species was not a significant factor in influencing contact angles in this analysis, either for dry silks (*F*_3,23_ = 1.7998, *p* = 0.1797) or for previously wetted silks (*F*_3,23_ = 2.0488, *p* = 0.139).

#### 3.2.2. Tilt angles

The volumes of water droplets that slipped off naturally spun silk samples did not differ between the four embiopteran species (ANOVA, *F*_3,1_ = 0.2476, *p* = 0.28) nor did they depend on the treatment of the silks (mean for dry silk = 63.8 ± 3.5 µl (s.e.) and for previously wetted = 68.1 ± 3.4 µl; *F*_3,1_ = 0.7241, *p* = 0.4049). A rose petal sample tested at the same time showed a lack of adhesion compared to the silk samples as reflected in the small droplet size (25 µl) that fell when the rose petal was tilted.

TA did not vary as a function of species (*F*_3,1_ = 1.36, *p* = 0.28) but, in contrast, the overall average TA for the four species for dry silk (85° ± 2°) was significantly higher than the average TA for previously wetted silk (74° ± 2°) (*F*_3,1_ = 10.45, *p* = 0.004). Because the water droplets tended to slip off at a lower TA when placed onto previously wetted silk samples, we conclude that adhesive properties due to the silk fibres' ability to pin water droplets [6] was partially lost when wetted silk transforms to a dry amorphous film.

#### 3.2.3. Accounting for chemical variations in webspinner silks

As shown in figure 4*a*, contact angles (shown on the left axis) are highest for silk species with the widest fibre diameters (shown on right axis). For example, *An. urichi* had highest average contact angle and largest average fibre diameter. The average static contact angles from *An. urichi* silk were the only results that overlapped with the rose petal. If previously exposed to water and then dried, all silks exhibited similar hydrophilic properties.

Fibre diameter and contact angles both increased in the following order (from lowest to highest): *P. trinitatis* ≅ *Ap. ceylonica* < *S. calypso* < *An. urichi.* These results suggest that fibre diameter roughly correlates with the contact angles reported here. The compositions of the lipid coatings of all four species were assumed to be similar to one another and to the lipid coatings of other arthropods silks, as previously suggested [5,6]. However, we considered possible differences in the hydrophobicity of the protein core material. The primary sequences of webspinner silk proteins contain highly repetitive amino acid units [7], which resemble those found in silk biopolymers from spiders [25] and silkworms [26]. Published amino acid sequences for *Ap. ceylonica*, *An. urichi* and *S. davisi* (a close relative of *S. calypso*) indicate that the glycine (Gly) and serine (Ser) content of silk from all three species was nearly identical, but the alanine (Ala) content varied, and was 5%, 10% and 17%, respectively [7]. As Ala is more hydrophobic than Ser, silks with higher percentage of Ala are predicted to be more hydrophobic. Our findings indicate that higher Ala content may contribute to higher contact angles. The amino acid content for *P. trinitatis* silk is reported here for the first time (electronic supplementary material, table S1). Ala content in *P. trinitatis* silk is about 2%, which was lower than the other three species studied. Based on Ala content in the primary sequence, the hydrophobicity of silks would increase in the following order: *P. trinitatis* < *Ap. ceylonica* < *An. urichi* < *S. calypso*. The measured static contact angles followed this overall trend, except the *An. urichi* silks had slightly higher contact angles than *S. calypso*, but they were not significantly different.

To ensure that protein secondary structure does not contribute to these observed differences in contact angles, we collected FTIR spectra of silks from all four species (electronic supplementary material, figure S4). The infrared absorbances in these spectra were used to describe the secondary structures, and found to be similar to one another. Regardless of Ala content, the silk from all four species exhibit predominately β-sheet structure [5]. These results are consistent with previously published studies of webspinner silks from *H. solieri*, *S. davisi*, *Archembia* sp., and *An. urichi* [8], which also indicated similarities in protein secondary structures. The differences in Ala content may not impact secondary structure, but may have a subtle effect on how β-sheets align in their three-dimensional structure. Previous studies suggested that Ala residues protruding from β-sheets may interlock into Gly residues from adjacent sheets [27]. Thus, fibres with higher Ala content may have proteins with molecular geometries that favour tightly locked core structures which exhibit decreased water penetration.

## 4. Conclusion

Our study made progress along three avenues of investigation into webspinner silk diversity: (i) developing methods for imaging and quantification, (ii) testing whether fibre diameter differences were correlated with insect size or evolutionary history, and (iii) exploring interactions between naturally spun silk and water, inspired by observations of natural colonies in the field. In this work, we used SEM and contact angle to characterize the physical properties of silk spun by webspinners directly onto graphite rods. Our procedures had two distinct advantages: (i) the silk samples were not disturbed by humans and should more accurately reflect natural diameters and water interaction properties, and (ii) the fibres were securely attached to the conductive graphite substrate, which reduced charging effects, and allowed collection of high quality SEM images without the use of metal coatings. Our access to nine species from five taxonomic families allowed us to test whether body length predicted fibre diameter or if diversity of silk fibres was more related to evolutionary history. The latter hypothesis gained support. Our survey adds significantly to the nanoscale description of webspinner silk, despite the relatively small sample size [12].

Using SEM, we observed that when silks were wetted with water, a film-like sheet results, which may indicate that proteins in the random coil conformation increase, similar to the structures formed when silks were soaked in methanol [5]. Previous studies suggested that organic solvent was required to dissolve the lipid coating in order to access the protein core. In this study, we expanded the protein–core model to include a description of how water interacts with the protein core in webspinner silk in the absence of organic solvent and show that exposure to water alone results in a change in macroscopic silk structure and wetting properties. Alternatively, exposure to water may remove metals that stabilize the β-sheet structure, as found in caddisfly silk, and when these metal cations are removed, water can penetrate rigid serine-phosphate regions and solvate the protein structure [28]. This hypothesis will be probed in future studies.

When wetted, webspinner silk transforms into a film and water drops slip off more easily. The biological significance is not currently understood, but we suspect that tropical arboreal webspinners may benefit from their silk's ability to shed water given the almost daily exposure to drenching rains [29]. From an applied point of view, as webspinner fibres become a film upon wetting, their molecular structures may inspire the design of a material which changes its physical properties when wetted. There is growing interest in studying nature-inspired materials to design new nanoscale surfaces with superhydrophobic properties for biomedical applications [30]. The principles of ‘pinned’ water droplets onto rough surfaces has previously been used to provide a set of ‘design criteria’ for creating a hydrophobic, yet water-adhering material [31]. However, more molecular insight from NMR and X-ray crystallography studies about the changes that occur in the three-dimensional structure upon exposure to water is required.

We reported fibre diameter for nine species from our laboratory cultures, but were able to quantify silk–water interactions for only four—those species that spin copious silk. Species that produce scant silk, such as *D. vandykei* and *H. solieri*, tend to dwell in crevices in bark, leaf litter and/or underground. They may produce silks with different characteristics than those living exposed on bark—such as reflected in the four species investigated herein. Determining how to collect silk from crevice-dwelling webspinners would increase our phylogenetic, as well as lifestyle, diversity and is worth pursuing. This is especially true given that previous work on *H. solieri* by Collin and co-workers [8] identified unique qualities in their silk proteins when compared to other webspinners. Finally, embiopterans come in a variety of body lengths beyond the range displayed by our current sample. Field collecting trips to Africa, South America and Southeast Asia would give us access to the largest webspinners (approaching 3 cm), and their silk might show qualities not detected in our more limited sample.

## Supplementary Material

Supporting Information

## Data accessibility

Data collected in this experiment, including amino acid content for *P. trinitatis* silk, mean fibre diameter listed in terms of species and adult female body length, contact and tilt angles, and sample sizes, are listed in tables included as electronic supplementary material.

## References

[1] Service RF 2017 Silken promises. Science 358, 293–294. (10.1126/science.358.6361.293) 29051360

[2] Vollrath F, Knight DP 2001 Liquid crystalline spinning of spider silk. Nature 410, 541–548. (10.1038/35069000) 11279484

[3] Pal RK, Kurland NE, Wang C, Kundu SC, Yadavalli VK 2015 Biopatterning of silk proteins for soft micro-optics. ACS Appl. Mater. Interfaces 7, 8809–8816. (10.1021/acsami.5b01380) 25853731

[4] Okada S, Weisman S, Trueman HE, Mudie ST, Haritos VS, Sutherland TD 2008 An Australian webspinner species makes the finest known insect silk fibers. Int. J. Biol. Macromol. 43, 271–275. (10.1016/j.ijbiomac.2008.06.007) 18619485

[5] Addison JB, Osborn Popp TM, Weber WS, Edgerly JS, Holland GP, Yarger JL 2014 Structural characterization of nanofiber silk produced by embiopterans (webspinners). RSC Adv. 4, 41 301–41 313. (10.1039/C4RA07567F) PMC4222186 25383190

[6] Osborn Popp TM, Addison JB, Jordan JS, Damle VG, Rykaczewski K, Chang SLY, Stokes GY, Edgerly JS, Yarger JL 2016 Surface and wetting properties of embiopteran (webspinner) nanofiber silk. Langmuir 32, 4681–4687. (10.1021/acs.langmuir.6b00762) 27062909

[7] Collin MA, Edgerly JS, Hayashi CY 2011 Comparison of fibroin cDNAs from webspinning insects: insight into silk formation and function. Zoology 114, 239–246. (10.1016/j.zool.2011.01.004) 21741226

[8] Collin MA, Camama E, Swanson BO, Edgerly JS, Hayashi CY 2009 Comparison of embiopteran silks reveals tensile and structural similarities across taxa. Biomacromolecules 10, 2268–2274. (10.1021/bm900449p) 19572641

[9] Dubitzky A, Melzer RR 1999 Untersuchung des Spinnvorgangs bei Haploembia solieri (Rambur) im REM. Nachrichtenblatt Bayer. Entomol. 48, 97–103.

[10] Büsse S, Hörnschemeyer T, Hohu K, McMillan D, Edgerly JS 2015 The spinning apparatus of webspinners—functional-morphology, morphometrics and spinning behaviour. Sci. Rep. 4, 9986 (10.1038/srep09986) 25950122 PMC4423565

[11] McMillan D, Hohu K, Edgerly JS 2016 Choreography of silk spinning by webspinners (Insecta: Embioptera) reflects lifestyle and hints at phylogeny. Biol. J. Linn. Soc. 118, 430–442. (10.1111/bij.12749)

[12] Miller KB, Hayashi CY, Whiting MF, Svenson GJ, Edgerly JS 2012 The phylogeny and classification of Embioptera (Insecta). Syst. Entomol. 37, 550–570. (10.1111/j.1365-3113.2012.00628.x)

[13] Silva LP, Rech EL 2013 Unravelling the biodiversity of nanoscale signatures of spider silk fibres. Nat. Commun. 4, 3014 (10.1038/ncomms4014) 24345771

[14] Schulte AJ, Droste DM, Koch K, Barthlott W 2011 Hierarchically structured superhydrophobic flowers with low hysteresis of the wild pansy (*Viola tricolor*)—new design principles for biomimetic materials. Beilstein J. Nanotechnol. 2, 228–236. (10.3762/bjnano.2.27) 21977435 PMC3148064

[15] Bhushan B, Nosonovsky M 2010 The rose petal effect and the modes of superhydrophobicity. Phil. Trans. R. Soc. A 368, 4713–4728. (10.1098/rsta.2010.0203) 20855317

[16] Kalyaanamoorthy S, Minh BQ, Wong TKF, von Haeseler A, Jermiin LS 2017 ModelFinder: fast model selection for accurate phylogenetic estimates. Nat. Methods 14, 587–589. (10.1038/nmeth.4285) 28481363 PMC5453245

[17] Nguyen L-T, Schmidt HA, von Haeseler A, Minh BQ 2015 IQ-TREE: a fast and effective stochastic algorithm for estimating maximum-likelihood phylogenies. Mol. Biol. Evol. 32, 268–274. (10.1093/molbev/msu300) 25371430 PMC4271533

[18] R Core Team. 2017 R: A language and environment for statistical computing. R Foundation for Statistical Computing. See https://www.r-project.org/ (accessed 4 January 2018).

[19] Paradis E, Claude J, Strimmer K 2004 APE: Analyses of Phylogenetics and Evolution in R language. Bioinformatics 20, 289–290. (10.1093/bioinformatics/btg412) 14734327

[20] Revell LJ 2012 phytools: an R package for phylogenetic comparative biology (and other things): phytools: R package. Methods Ecol. Evol. 3, 217–223. (10.1111/j.2041-210X.2011.00169.x)

[21] Tung Ho L, Ané C 2014 A linear-time algorithm for Gaussian and non-Gaussian trait evolution models. Syst. Biol. 63, 397–408. (10.1093/sysbio/syu005) 24500037

[22] Butler MA, King AA 2004 Phylogenetic comparative analysis: a modeling approach for adaptive evolution. Am. Nat. 164, 683–695. (10.1086/426002) 29641928

[23] Feng L, Zhang Y, Xi J, Zhu Y, Wang N, Xia F, Jiang L 2008 Petal effect: a superhydrophobic state with high adhesive force. Langmuir 24, 4114–4119. (10.1021/la703821h) 18312016

[24] Schneider CA, Rasband WS, Eliceiri KW 2012 NIH Image to ImageJ: 25 years of image analysis. Nat. Methods 9, 671–675. (10.1038/nmeth.2089) 22930834 PMC5554542

[25] Lewis RV 2006 Spider silk: ancient ideas for new biomaterials. Chem. Rev. 106, 3762–3774. (10.1021/cr010194g) 16967919

[26] Lefèvre T, Rousseau M-E, Pézolet M 2007 Protein secondary structure and orientation in silk as revealed by Raman spectromicroscopy. Biophys. J. 92, 2885–2895. (10.1529/biophysj.106.100339) 17277183 PMC1831708

[27] Lewin M 2007 Handbook of fiber chemistry, 3rd edn Boca Raton, FL: CRC Press.

[28] Addison JB, Weber WS, Mou Q, Ashton NN, Stewart RJ, Holland GP, Yarger JL 2014 Reversible assembly of β-sheet nanocrystals within caddisfly silk. Biomacromolecules 15, 1269–1275. (10.1021/bm401822p) 24576204 PMC4096554

[29] Edgerly JS 1997 Life beneath silk walls: a review of the primitively social Embiidina. In The evolution of social behavior in insects and arachnids (eds Choe JC, Crespi BJ), pp. 14–25. Cambridge, UK: Cambridge University Press.

[30] Falde EJ, Yohe ST, Colson YL, Grinstaff MW 2016 Superhydrophobic materials for biomedical applications. Biomaterials 104, 87–103. (10.1016/j.biomaterials.2016.06.050) 27449946 PMC5136454

[31] Extrand CW 2002 Model for contact angles and hysteresis on rough and ultraphobic surfaces. Langmuir 18, 7991–7999. (10.1021/la025769z)

