# Supplementary material for: Structural and wetting properties of nature's finest silks (order Embioptera)
Source: R Soc Open Sci. 2018 Sep 12;5(9):180893. doi: 10.1098/rsos.180893 (PMC6170577; doi:10.1098/rsos.180893)
Supplement: Supporting Information [file rsos180893supp1.pdf]

Supporting Information for

Structural and Wetting Properties of Nature's Finest Silks (Order Embioptera)

Grace Y. Stokes,[a] Evangelea N. DiCicco,[b] Trevor J. Moore,[a] Vivian C. Cheng,[c] Kira Y. Wheeler,[b] John Soghigian,[d] Richard P. Barber, Jr.,[b] Janice S. Edgerly[c]*

[a]Dept. of Chemistry & Biochemistry,

[b]Dept. of Physics and Center for Nanostructures,

[c]Dept. of Biology,

500 El Camino Real, Santa Clara University, Santa Clara, CA 95053

[d]Dept. of Environmental Sciences, The Connecticut Agricultural Experiment Station

Table of Contents **Page**

1. Silk fiber diameter versus insect body length	S1
2. Diameters of scooped versus spun silk	S2
3. Photograph of the apparatus used to determine tilt angle	S3
4. FTIR spectra of dry silk from four webspinner species (with peak assignments)	S4
5. Zoomed-in SEM images of metalized silks demonstrate diversity of silken sheet structures	S5
6. Table of alanine content for *P. trinitatis*	S6
7. Table of fiber diameter, insect size, and phylogenetic relatedness of species	S6
8. Tables of contact angle and tilt angle data	S7
9. Supporting information references	S7

Fig. S1. Silk fiber diameter versus average adult female body length. Data are found in Table S2.

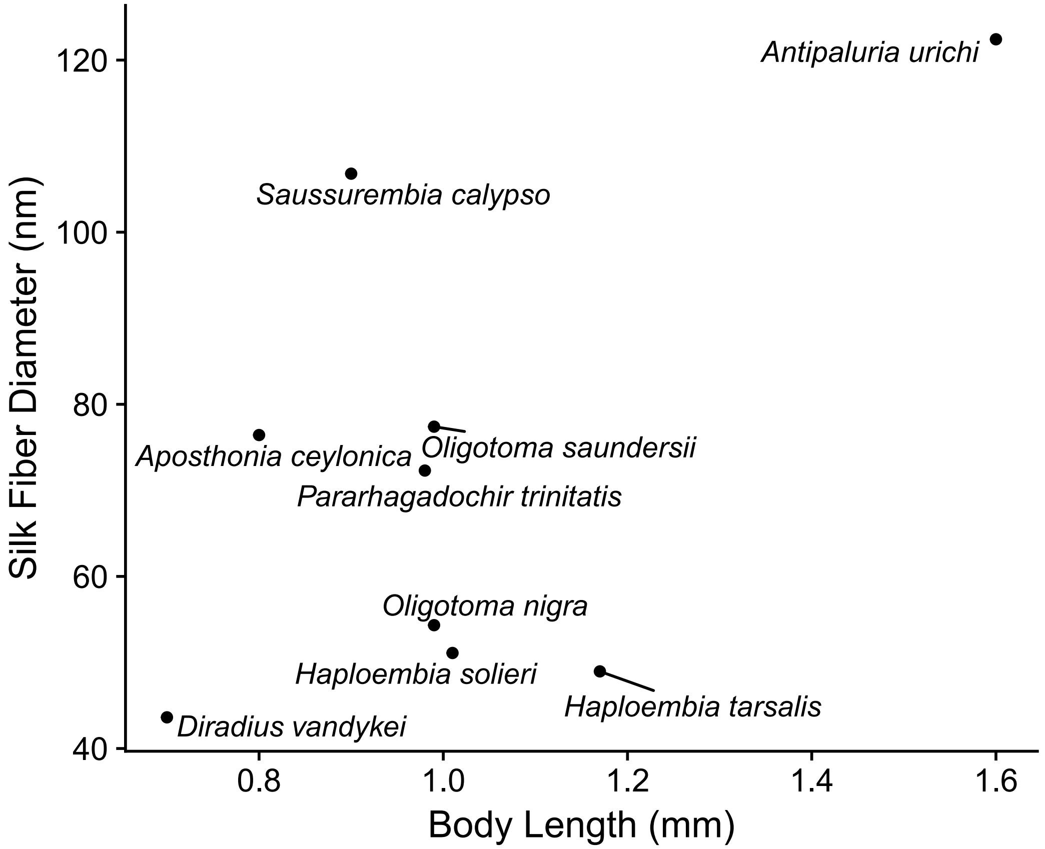

Fig. S2. Diameters of scooped (A) and naturally spun (B) silk samples adhered to graphite rods (2.4 mm diameter for the small insects or 6.4 mm diameter for the larger insects spinning the silk), used for scanning electron microscopy. Diamond shapes indicate mean (middle line), one standard error (SE) of the mean above and below (top and bottom lines) and 95% confidence around the mean (top and bottom tips of the diamond). Individual points show raw data. The Tukey-Kramer circles indicate the overlap amongst all data sorted by species. Silk diameters were significantly different between species for Scooped ($F_{7, 61} = 15.47$, $P < 0.0001$) and for Spun ($F_{8,88} = 51.48$, $P < 0.001$) but the extreme overlap for the scooped samples indicated that the differences were compressed compared to that found in naturally spun silk. Data for scooped *Haploembia tarsalis* is missing because these webspinners spin so little silk, it was not possible to scoop it out of the culture.

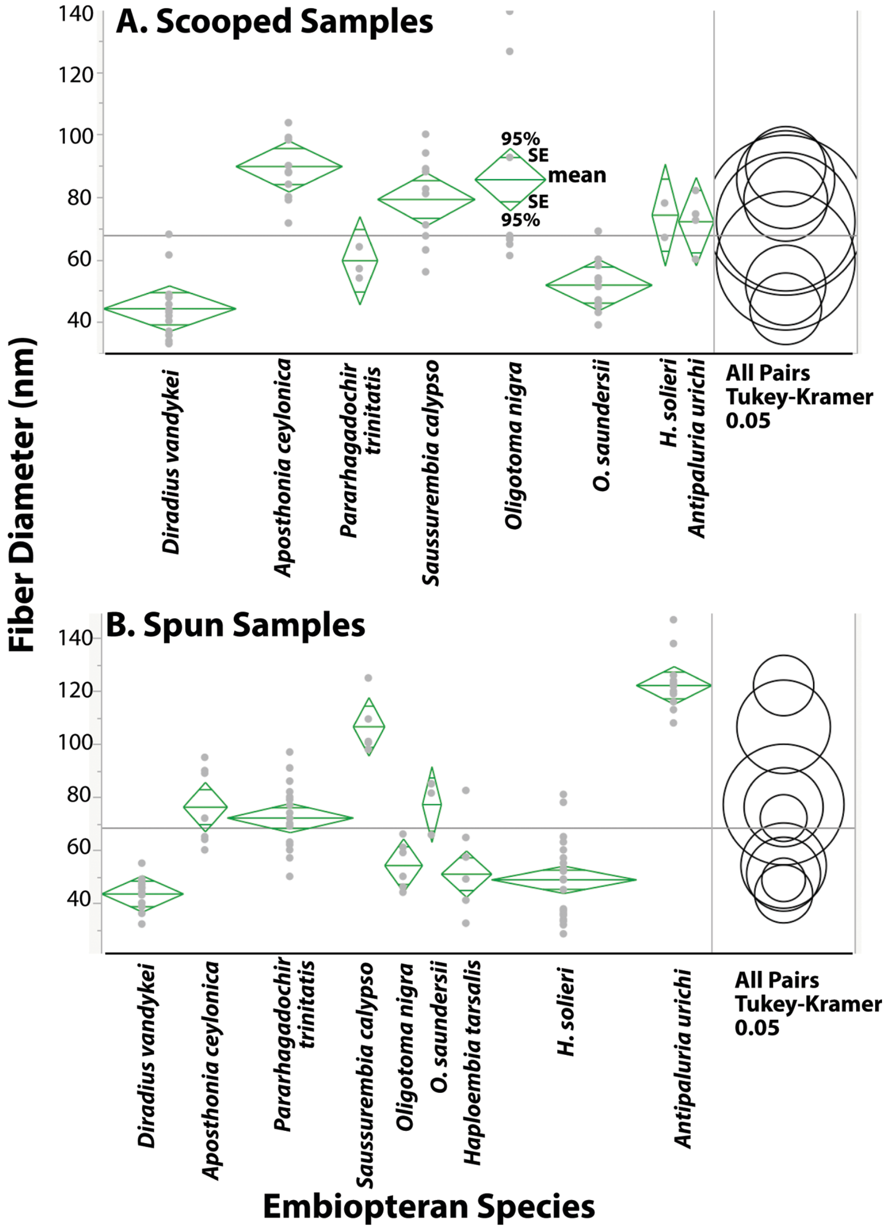

Fig. S3. Photograph of the apparatus used to determine tilt angle. Constructed by Stanley Tharaud.

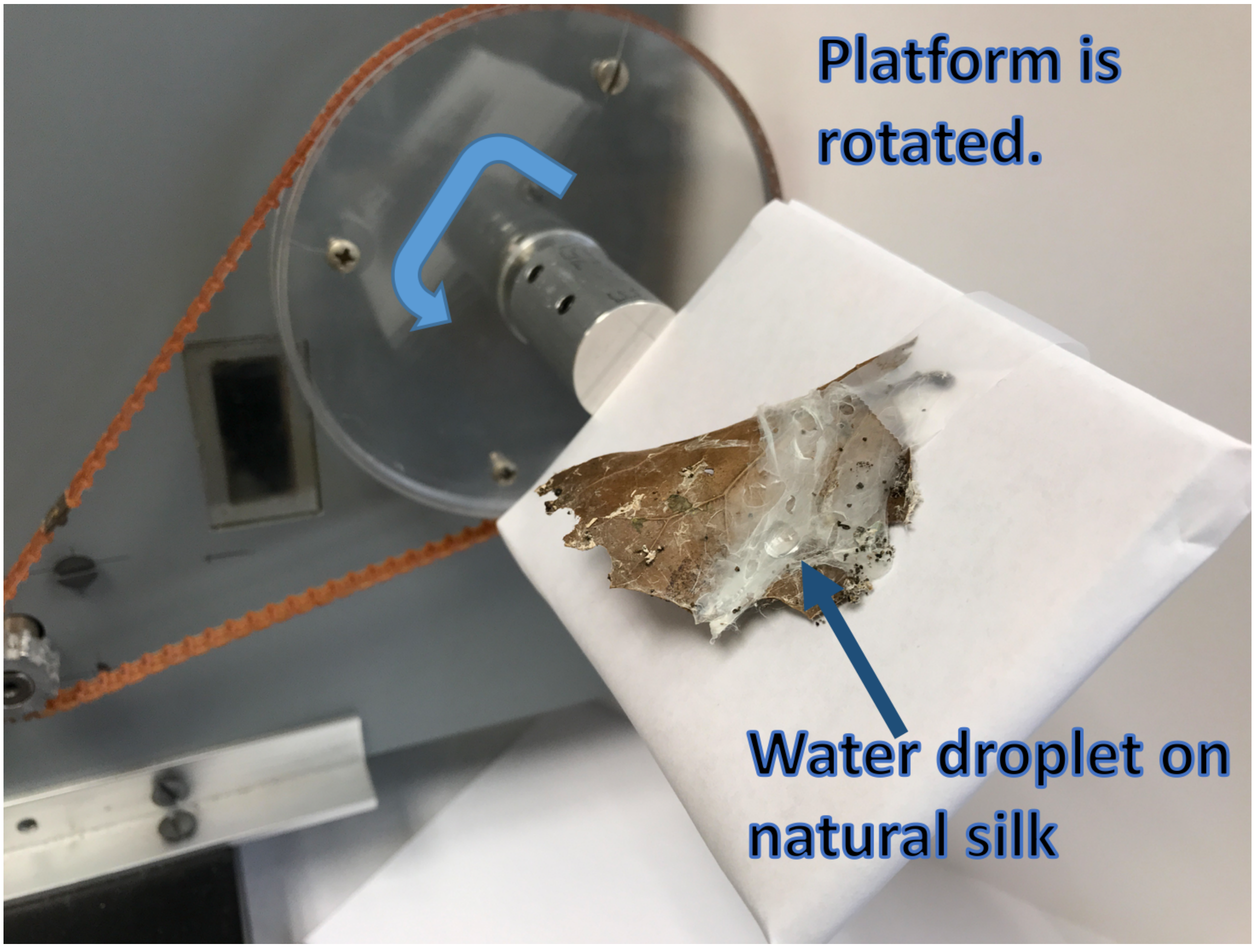

Fig. S4. FTIR spectra of dry silk from four webspinner species. Following Addison and co-workers,[1] the following assignments were made: Amide I (1624 cm^{-1}) and Amide II (1512 cm^{-1}) absorbances suggest that secondary structure is predominantly β-sheet. Amide III (1236 cm^{-1}) absorbance may be attributed to either β-sheet or random coil structures.

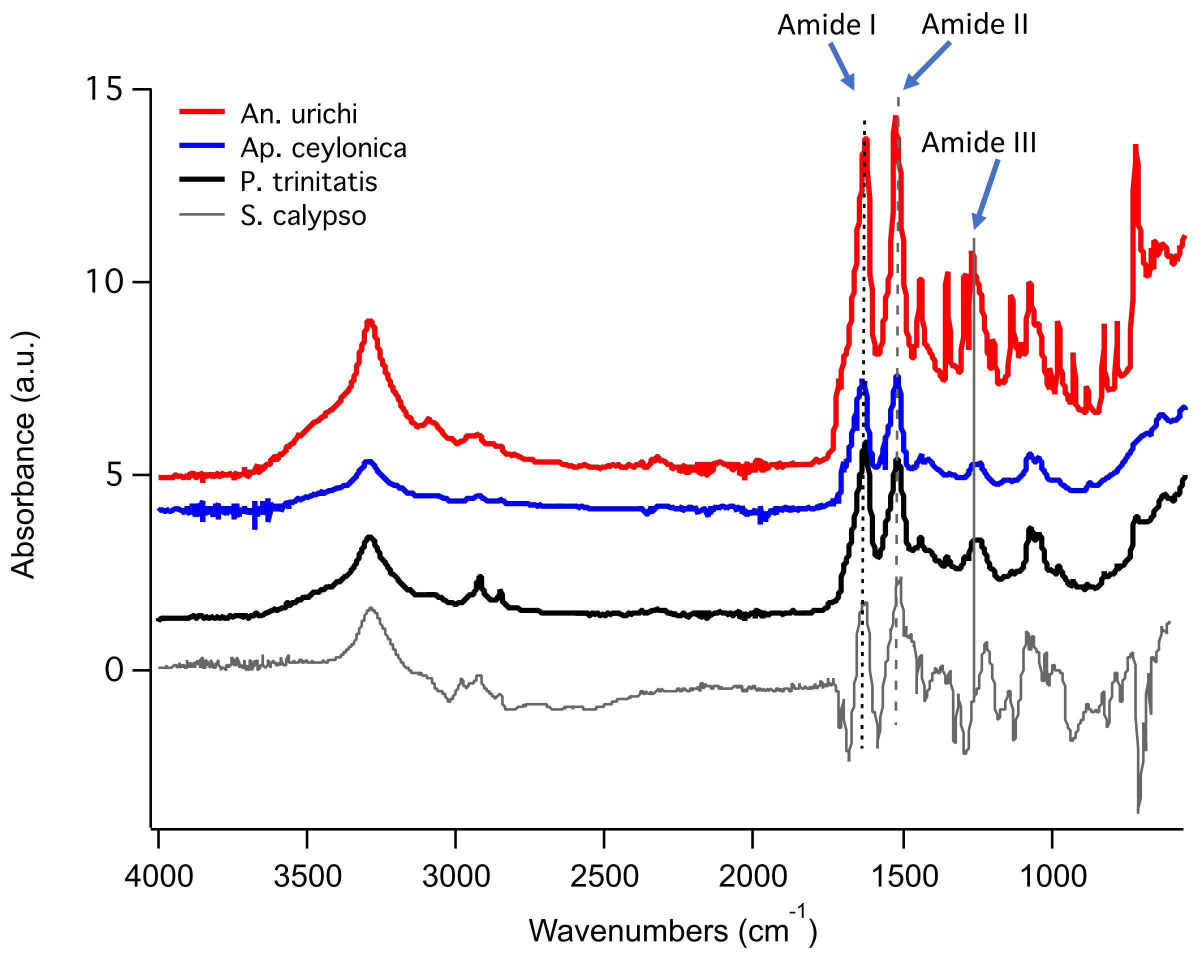

Fig. S5. Representative scanning electron microscopy (SEM) images of silks from **(A)** *An. urichi* and **(B)** *Ap. ceylonica* show the diversity and differences in naturally-spun silk. These zoomed in SEM images of metallized silken sheets show how surface area may vary, which can impact contact angles. Notice the 2-3 micron sized holes in **(A)** and the parallel fibers in **(B)**.

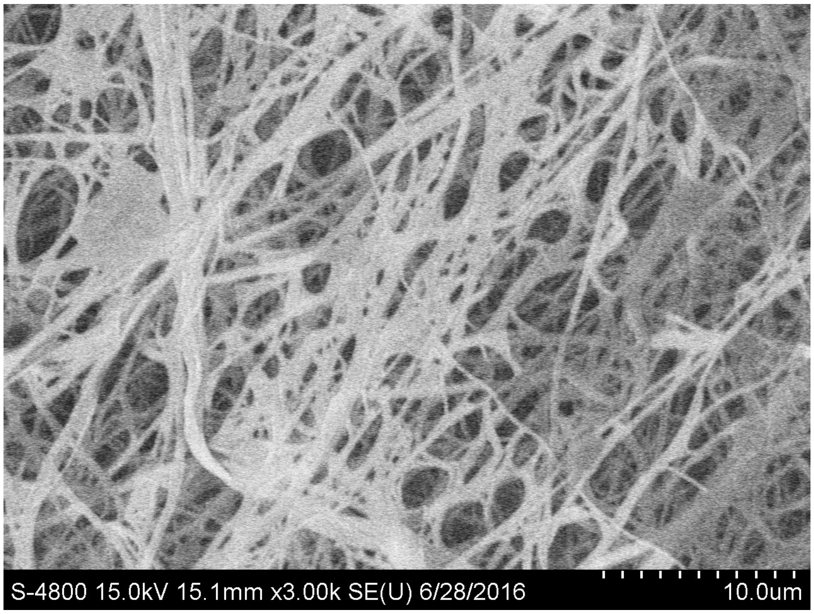

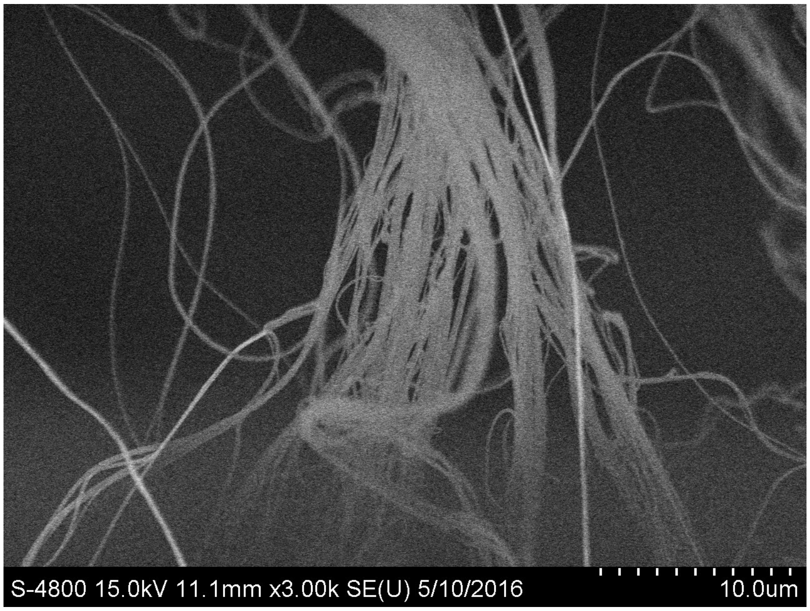

Table S1. Amino acid content (percentages) of *P. trinitatis* silk. Mean and standard deviations (SD) are determined from three different silk samples which were submitted to University of Nevada Reno Protein Core Facility for amino acid analysis.

Amino Acid	% (mean	± SD)
Gly	47.28	± 1.20
Ser	35.78	± 1.58
Asx	4.08	± 0.29
Glx	3.77	± 0.58
Ala	2.32	± 0.35
His	1.08	± 0.29
Val	0.87	± 0.10
Tyr	0.84	± 0.12
Pro	0.79	± 0.16
Thr	0.67	± 0.20
Lys	0.62	± 0.17
Leu	0.57	± 0.14
Ile	0.52	± 0.15
Phe	0.46	± 0.17
Arg	0.30	± 0.12
Met	0.07	± 0.06
Cys	0.00	± 0.00

Table S2. Fiber diameter, adult female body length, number of fibers and number of samples shown in parentheses

Species (listed in order of phylogenetic relatedness)	Fiber diameter (mean ± SE) (nm) (number of fibers measured)		Adult female body length (mean ± SE) (cm) (number of samples)	
Oligotoma saundersii	77.4	±5.9 (15)	0.99	±0.04 (7)
Oligotoma nigra	54.3	±3.6 (14)	1.00	±0.02 (7)
Aposthonia ceylonica	76.4	±5.5 (19)	0.95	±0.05 (10)
Haploembia tarsalis	49.0	±3.1 (23)	1.26	±1.17 (6)
Haploembia solieri	51.1	±5.8 (11)	1.01	±0.03 (10)
Diradius vandykei	43.6	±1.7 (28)	0.70	NA (1)
Pararhagadochir trinitatis	72.3	±2.7 (24)	1.02	±0.02 (10)
Antipaluria urichi	122.4	±3.2 (16)	1.59	±0.01 (67)
Saussurembia calypso	106.8	±5.0 (16)	0.60	±0.02 (17)

Table S3. Contact angles (initial, dry and previously wet), with sample sizes in parentheses (paired tests were conducted on single sample in two different spots: one dry and one previously wetted and then dried before testing again)

		Pair Test Contact Angles (mean ° ± SE)	
Species	Initial contact angle (mean ° ± SE) (number of samples measured)	Dry Silk Spot (mean ° ± SE)	Rewetted Silk Spot (mean ° ± SE)
Antipaluria urichi	122 ± 3 (15)	123 ± 6 (5)	77 ± 8
Aposthonia ceylonica	116 ± 2 (15)	115 ± 3 (4)	47 ± 11
Pararhagadochir trinitatis	112 ± 2 (15)	101 ± 3 (3)	74 ± 2
Saussurembia calypso	120 ± 3 (14)	119 ± 13 (3)	54 ± 14
PDMS	112 ± 2 (5)		
Rose petal	134 ± 3 (5)		

Table S4. Tilt angles (dry and previously wet) with associated drop volumes (n = 3 samples per species for dry and for previously wetted except for the dry silk for *An. urichi* with n = 4 samples)

	Dry Silk Samples (mean° ± SE)		Previously Wet Silk Samples (mean ° ± SE)	
Species	Tilt Angle (mean ° ± SE)	Drop Volume (mean µL ± SE)	Tilt Angle (mean ° ± SE)	Drop Volume (mean µL ± SE)
Antipaluria urichi	90 ± 1	61.2 ±3.3	70 ± 6	72.5 ± 8.8
Aposthonia ceylonica	81 ± 5	60.0 ±5.7	73 ± 6	68.3 ± 4.4
Pararhagadochir trinitatis	87 ± 3	66.7 ±6.7	82 ± 3	60 ± 3
Saussurembia calypso	81 ± 5	66.7 ±9.3	71 ± 5	70 ± 10

Supporting Information References

(1) Addison, J. B.; Osborn Popp, T. M.; Weber, W. S.; Edgerly, J. S.; Holland, G. P.; Yarger, J. L. Structural Characterization of Nanofiber Silk Produced by Embiopterans (Webspinners). *RSC Adv* **2014**, *4* (78), 41301–41313.
